# Impact of Contrast-Enhanced Computed Tomography on the Management of Postpartum Hemorrhage with Extravasation

**DOI:** 10.3390/jcm15145642

**Published:** 2026-07-18

**Authors:** Suk Hwan Hyun, Byung Hun Kang, Hae Ri Kim, Ye Won Jung, Won Kyo Shin, Soo Youn Song, Jae Sung Choi, Young Bok Ko, Mina Lee, Mia Park, You Jin Kim, Hyeon Jin Na, Yeo Kyeong Bae, Heon Jong Yoo

**Affiliations:** 1 Department of Obstetrics and Gynecology, Chungnam National University Sejong Hospital, 20 Bodeum 7-ro, Sejong 30099, Republic of Korea; neo1714@cnuh.co.kr (S.H.H.); yo0118@cnuh.co.kr (H.R.K.); wonyberry@cnuh.co.kr (Y.W.J.); bluered120@cnuh.co.kr (W.K.S.); sysong@cnuh.co.kr (S.Y.S.); cjs570@cnuh.co.kr (J.S.C.); 2 Department of Obstetrics & Gynecology, Chungnam National University School of Medicine, 266 Munhwa-ro, Jung-gu, Daejeon 35015, Republic of Korea; missinglime@cnuh.co.kr (B.H.K.); koyoung27@cnuh.co.kr (Y.B.K.); minari73@cnuh.co.kr (M.L.); mia86@cnuh.co.kr (M.P.); niguemai@cnuh.co.kr (Y.J.K.); nhj0136@cnuh.co.kr (H.J.N.); byk006@cnuh.co.kr (Y.K.B.); 3 Department of Obstetrics and Gynecology, Chungnam National University Hospital, 282 Munhwa-ro, Jung-gu, Daejeon 35015, Republic of Korea; 4 Department of Internal Medicine, Chungnam National University School of Medicine, 266 Munhwa-ro, Jung-gu, Daejeon 35015, Republic of Korea

**Keywords:** contrast-enhanced CT, extravasation, postpartum hemorrhage, surgical management, shock index

## Abstract

**Background:** This study aimed to investigate the association of contrast extravasation (EV) detected on contrast-enhanced computed tomography (CE-CT) with the clinical characteristics, management, and outcomes of patients with postpartum hemorrhage (PPH). **Methods**: We retrospectively analyzed 51 patients with PPH who underwent CE-CT between August 2013 and July 2024. Patients were classified into EV (−) group (*n* = 28) and EV (+) group (*n* = 23). Baseline characteristics, management strategies, and clinical outcomes were compared. **Results:** The proportion of patients with a shock index (SI) ≥ 1.0 was significantly higher in the EV (+) group (10.7% vs. 39.1%, *p* = 0.017). Surgical intervention was more frequently required in the EV (+) group (10.7% vs. 56.5%, *p* = 0.001), whereas conservative management alone was more frequently successful in the EV (−) group (89.3% vs. 43.5%, *p* = 0.001). The EV (+) group showed higher rates of massive blood loss (> 2000 mL, 3.6% vs. 34.8%, *p* = 0.009), longer hospital stays (4.61 ± 2.61 vs. 6.88 ± 3.78 days, *p* = 0.034), and more frequent intensive care unit (ICU) admission (10.7% vs. 34.8%, *p* = 0.039). Patients in the EV (+) group had higher transfusion requirements (*p* < 0.005) and increased rates of disseminated intravascular coagulation (7.1% vs. 30.4%, *p* = 0.032). **Conclusions**: The presence of EV on CE-CT in PPH was associated with more severe clinical features, including greater transfusion requirements, increased rates of surgical intervention, ICU admission, and disseminated intravascular coagulation (DIC). Additionally, a higher proportion of patients with EV had a shock index ≥ 1.0, indicating early hemodynamic compromise.

## 1. Introduction

Postpartum hemorrhage (PPH) is a leading cause of maternal morbidity and mortality worldwide, occurring in approximately 1% to 10% of all deliveries [1]. Despite advances in obstetric care, PPH continues to present a significant clinical challenge and therefore requires prompt recognition and effective management to reduce adverse outcomes [2]. Uterine atony remains the most common cause of PPH, but other etiologies such as trauma, retained products of conception, and coagulopathy must also be considered. Early identification and prompt management of PPH are critical for preventing serious complications, including disseminated intravascular coagulation (DIC), organ failure, and death [3]. Conventional techniques such as ultrasonography may reveal clots and retained products of conception but often provide limited information regarding the precise source of bleeding, especially in extrauterine sites [4].

In recent years, contrast-enhanced computed tomography (CE-CT) has been increasingly utilized as a valuable modality for evaluating PPH [5,6,7,8]. CE-CT enables rapid and accurate localization of active bleeding sites, particularly in patients with hemodynamic instability or when conventional assessments fail to identify the source [5]. Importantly, the presence of contrast extravasation (EV) on CE-CT has been associated with more severe hemorrhagic conditions and a higher likelihood of requiring invasive interventions such as uterine artery embolization (UAE) or surgery [6]. Several studies have shown that EV identified on CE-CT demonstrates high concordance with angiographic findings and can serve as a predictive marker for the need for hemostatic procedures in PPH [6,7,8]. As a result, CE-CT is increasingly integrated into clinical treatment algorithms for refractory PPH in tertiary care settings [8]. Despite its growing clinical utility, the specific association of EV observed on CE-CT on clinical decision-making and outcomes in PPH remains underexplored. Therefore, this study aimed to investigate the association of contrast extravasation detected on contrast-enhanced computed tomography (CE-CT) with the clinical characteristics, management, and outcomes of patients with postpartum hemorrhage (PPH).

## 2. Materials and Methods

**Figure 1:**
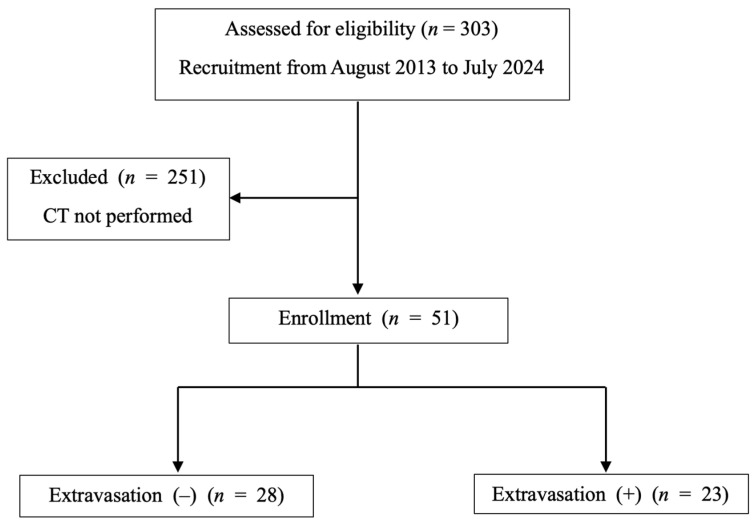
Flowchart of the study participant selection. CT = Computed Tomography.

This retrospective cohort study analyzed the clinical records of 303 patients treated for PPH at Chungnam National University Hospital and Chungnam National University Sejong Hospital between August 2013 and July 2024. Among them, 51 patients underwent CE-CT during the acute phase of hemorrhage for further evaluation and were included in this study (Figure 1). CE-CT was performed selectively at the discretion of the attending obstetrician when additional imaging was considered clinically necessary. The study was approved by the Institutional Review Board of Chungnam National University Sejong Hospital (IRB No. 2025-07-031), and the need for informed consent was waived due to its retrospective nature.

**Figure 2:**
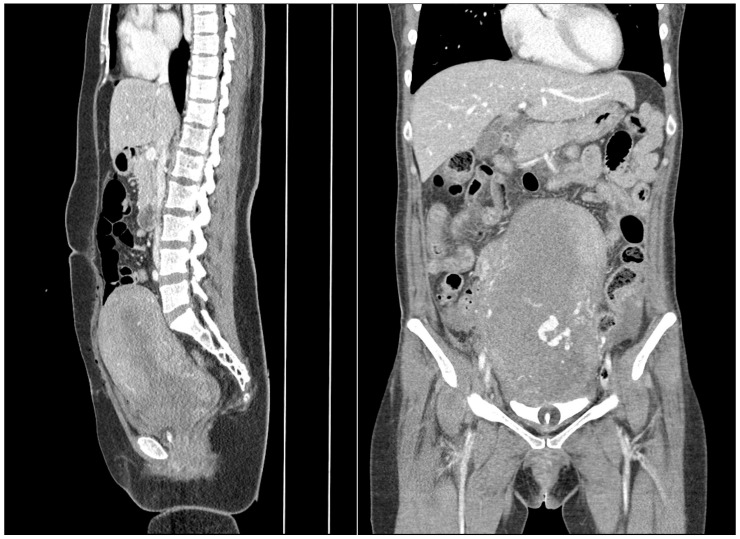
Sagittal (**left**) and coronal (**right**) contrast-enhanced computed tomography (CE-CT) images of two patients with postpartum hemorrhage (PPH). (**Left**) A case without contrast extravasation on CE-CT. The patient was successfully managed with medical treatment including uterotonics and hemostatic agents without the need for surgical intervention. (**Right**) A case showing definite contrast extravasation in the uterine cavity. Despite initial conservative treatment including uterotonics, hemostatic agents, and uterine artery embolization (UAE), the patient failed to respond and ultimately required surgical management with hysterectomy.

PPH was defined as blood loss ≥500 mL regardless of the mode of delivery, in accordance with recent clinical guidelines [2]. Patients were categorized into two groups: those without EV on CE-CT (EV (−) group, *n* = 28) and those with EV on CE-CT (EV (+) group, *n* = 23). All CE-CT studies were performed using a multiphase protocol, and the presence of EV was assessed by experienced radiologists who were blinded to clinical outcomes. Representative CE-CT images depicting the presence or absence of contrast EV in each group are shown in Figure 2.

Clinical data were collected retrospectively from electronic medical records of the two participating institutions. Variables included demographic and obstetric characteristics such as age, body mass index (BMI), presence of hypertension and diabetes, parity (primipara or multipara), number of fetuses (singleton or multiple), gestational age at delivery (term or preterm), mode of delivery (vaginal or cesarean), history of previous cesarean section, place of delivery (inborn or outborn), and timing of PPH onset (primary or secondary). Initial assessments included laboratory values such as white blood cell count, hemoglobin, hematocrit, platelet count, and lactic acid level, as well as vital signs including systolic and diastolic blood pressure, heart rate, and body temperature. Shock index (SI), defined as the ratio of heart rate to systolic blood pressure, was calculated, and patients were stratified based on whether the SI was ≥1.0. This SI threshold was selected in accordance with the current World Health Organization (WHO) guideline, which considers a shock index ≥1 as an abnormal hemodynamic sign [9].

Management strategies were classified as either conservative or surgical. Conservative treatment was considered effective when hemostasis was achieved without additional procedures; otherwise, cases were considered to have required conversion to surgical management. Surgical procedures were categorized as immediate interventions or those performed following failed conservative treatment. Conservative management was defined as uterus-preserving treatment, including medical therapy, uterine curettage, and uterine artery embolization (UAE). Surgical management was defined as operative procedures performed in the operating room under general or regional anesthesia.

Clinical outcomes were evaluated, including estimated blood loss categorized into 500–1000 mL, 1000–2000 mL, and >2000 mL. Estimated blood loss was recorded according to the routine clinical assessment documented in the electronic medical record. Additional outcome measures included length of hospital stay and intensive care unit (ICU) admission. Transfusion requirements were assessed by the number of units administered for packed red blood cells, fresh frozen plasma, and platelet concentrates. Complications were recorded, including DIC, acute renal failure, fever, hypertension, and pulmonary, cardiac, or cerebral complications, as well as other events such as deep vein thrombosis and Sheehan syndrome.

Comparisons between the EV (−) and EV (+) groups were made using the *t*-test for normally distributed continuous variables or the Mann–Whitney U test when normality was not met. For categorical variables, the chi-square test or Fisher’s exact test was used when the expected frequency within any cell was less than 5. Given the exploratory nature of the analyses, no adjustment for multiple comparisons was performed. Statistical analyses were performed using SPSS software (version 20.0; IBM Corp., Armonk, NY, USA). Statistical significance was defined as *p* < 0.05.

## 3. Results

Table 1 summarizes the obstetric and baseline characteristics of the patients. There were no statistically significant differences between the EV (−) and EV (+) groups in age (34.79 ± 4.26 vs. 34.30 ± 4.41 years, *p* = 0.695), parity (1.54 ± 0.88 vs. 1.61 ± 0.65, *p* = 0.489), presence of hypertension (10.7% vs. 4.3%, *p* = 0.405), diabetes (10.7% vs. 4.3%, *p* = 0.405), number of fetuses (singleton: 89.3% vs. 91.3%, *p* = 0.811), term delivery (96.4% vs. 95.7%, *p* = 0.888), mode of delivery (vaginal: 39.3% vs. 47.8%, *p* = 0.540), previous cesarean delivery (25.0% vs. 13.0%, *p* = 0.289), place of delivery (inborn: 92.9% vs. 87.0%, *p* = 0.485), and onset of PPH (primary: 67.9% vs. 56.5%, *p* = 0.405). However, BMI was significantly higher in the EV (−) group than in the EV (+) group (26.64 ± 3.90 vs. 23.96 ± 3.88 kg/m^2^, *p* = 0.021).

Initial laboratory and vital sign results are presented in Table 2. There were no statistically significant differences between the EV (−) and EV (+) groups in WBC count (16.21 ± 5.70 vs. 19.34 ± 8.45 × 10^3^/µL, *p* = 0.123), hemoglobin (9.99 ± 2.02 vs. 9.53 ± 1.92 g/dL, *p* = 0.407), hematocrit (29.22 ± 5.70 vs. 27.90 ± 5.51%, *p* = 0.406), platelet count (219.71 ± 86.91 vs. 232.52 ± 115.28 × 10^3^/µL, *p* = 0.653), or lactic acid level (2.65 ± 1.65 vs. 2.65 ± 1.22 mmol/L, *p* = 0.650). Regarding vital signs, no statistically significant differences were observed in systolic blood pressure (118.25 ± 23.73 vs. 111.87 ± 24.69 mmHg, *p* = 0.353), diastolic blood pressure (75.04 ± 19.44 vs. 68.70 ± 18.55 mmHg, *p* = 0.130), or body temperature (37.21 ± 0.92 vs. 37.00 ± 0.66 °C, *p* = 0.369). Heart rate showed a borderline significant difference (89.57 ± 14.75 vs. 101.61 ± 27.55 bpm, *p* = 0.052). The difference in mean SI between the EV (−) and EV (+) groups was borderline significant (0.78 ± 0.22 vs. 0.95 ± 0.37, *p* = 0.062). However, a significantly higher proportion of patients in the EV (+) group had a SI ≥ 1.0 (10.7% vs. 39.1%, *p* = 0.017), suggesting greater early hemodynamic compromise.

Table 3 shows the distribution of causes of PPH. In the EV (−) group, uterine atony was the most common cause (64.3%), followed by trauma (21.4%) and tissue-related causes (14.3%). In the EV (+) group, uterine atony (47.8%) was also the most frequent cause, followed by trauma (30.4%) and tissue-related causes (21.7%). No thrombin-related causes were reported in either group. The overall distribution of PPH etiologies did not differ significantly between the two groups (*p* = 0.496).

Management strategies are summarized in Table 4. The EV (+) group had significantly higher rates of surgical management than the EV (−) group (10.7% vs. 56.5%, *p* = 0.001), including immediate surgical intervention (3.6% vs. 43.5%) and conversion from conservative treatment (7.1% vs. 13.0%). Conversely, the EV (−) group underwent conservative management more frequently than the EV (+) group (89.3% vs. 43.5%). Types of surgical procedures between the EV (−) and EV (+) groups included laceration repair (0% vs. 8.7%), placental removal (0% vs. 4.3%), hysterectomy (7.1% vs. 30.4%), and other surgical procedures (3.6% vs. 13.0%). Types of conservative management between the two groups included uterotonics (67.9% vs. 56.5%, *p* = 0.405), hemostatic agents (57.1% vs. 52.2%, *p* = 0.723), curettage (3.6% vs. 0%, *p* = 0.365), gauze packing (10.7% vs. 17.4%, *p* = 0.495), intrauterine balloon tamponade (21.4% vs. 13.0%, *p* = 0.439), and UAE (28.6% vs. 47.8%, *p* = 0.157). There were no statistically significant differences in the types of conservative management between the groups.

Table 5 presents clinical outcomes and complications. The EV (+) group had significantly greater estimated blood loss than the EV (−) group, with more patients experiencing >2000 mL (3.6% vs. 34.8%, *p* = 0.009). The length of hospital stay was longer in the EV (+) group (4.61 ± 2.61 vs. 6.88 ± 3.78 days, *p* = 0.034), and ICU admission was more frequent (10.7% vs. 34.8%, *p* = 0.039). Transfusion requirements were significantly higher in the EV (+) group for packed red blood cells (2.54 ± 2.21 vs. 7.61 ± 7.59 units, *p* = 0.002), fresh frozen plasma (1.75 ± 2.33 vs. 7.43 ± 8.34 units, *p* = 0.002), and platelet concentrates (1.71 ± 4.70 vs. 11.26 ± 16.02 units, *p* = 0.005). Regarding complications, DIC was significantly more frequent in the EV (+) group (7.1% vs. 30.4%, *p* = 0.032). Borderline significant increases were noted for hypertension (0% vs. 13.0%) and pulmonary complications (0% vs. 13.0%) in the EV (+) group (both *p* = 0.051), suggesting a possible trend toward increased systemic complications. Other complications, including acute renal failure (0% vs. 0%), fever (21.4% vs. 13.0%, *p* = 0.439), cardiac complications (0% vs. 4.3%, *p* = 0.270), cerebral complications (0% vs. 4.3%, *p* = 0.270), Sheehan syndrome (0% vs. 0%), and deep vein thrombosis (0% vs. 0%), showed no significant differences between the groups.

A multivariable logistic regression analysis was performed to determine whether contrast extravasation was independently associated with surgical management after adjustment for BMI and shock index ≥ 1.0 (Table 6). Contrast extravasation remained significantly associated with surgical management after adjustment for BMI and shock index. (adjusted OR, 7.444; 95% CI, 1.582–35.033; *p* = 0.011), whereas BMI and shock index ≥1.0 were not independently associated with surgical management.

## 4. Discussion

This study revealed that the presence of EV was associated with more frequent surgical intervention, longer hospital stays, more frequent ICU admission, greater transfusion requirements, and increased rates of DIC. The EV (+) group showed a consistent pattern of more severe clinical course. The EV (+) group also had a significantly higher proportion of patients with a SI ≥ 1.0, suggesting early hemodynamic compromise. Further studies are needed to confirm these findings.

Previous studies consistently demonstrate that the presence of EV on CE-CT is a strong and reliable predictor of the need for aggressive management in patients with Previous Studies have consistently demonstrated that EV on CE-CT is associated with active bleeding, greater blood loss, increased transfusion requirement, and a higher likelihood of uterine artery embolization or other invasive interventions [4,6,7,8,10,11,12]. Our findings are in agreement with these reports, as patients with EV more frequently required surgical intervention and significantly greater transfusion support than those without EV. Importantly, beyond confirming these previously reported associations, our study further demonstrated that EV-positive patients experienced longer hospital stays, higher rates of intensive care unit admission, and more frequent disseminated intravascular coagulation. In addition, although the continuous shock index did not differ significantly between groups, the proportion of patients with a shock index ≥1.0 was significantly higher in the EV-positive group, suggesting that EV may also reflect early hemodynamic compromise. Taken together, these findings support the role of EV as an imaging marker associated with a more severe clinical course in postpartum hemorrhage rather than merely indicating the presence of active bleeding. These findings are summarized in Table 7.

The finding that the EV (+) group had a lower BMI than the EV (−) group contrasts with previous studies reporting an increased risk of atonic PPH in overweight and obese women [13,14] and a decreased risk in underweight women [15]. Obesity has been associated with impaired uterine contractility and dysfunctional labor, which may contribute to increased bleeding in obese women [16], whereas individuals with lower BMI may become hemodynamically unstable more rapidly due to reduced circulating blood volume, potentially amplifying the severity of hemorrhage once active bleeding begins [17]. These factors suggest that BMI may influence both the risk of PPH and the physiologic response once bleeding occurs. This may partly explain why lower BMI in the EV (+) group corresponded with more severe presentations. However, this finding should be interpreted with caution, as our retrospective study included only patients who underwent CE-CT based on clinical judgment, and selection bias may also have contributed to the observed association between lower BMI and EV (+). Further studies are needed to clarify how maternal BMI affects bleeding dynamics and the requirement for invasive management in severe PPH.

The SI, defined as the ratio of heart rate to systolic blood pressure, has long been used in trauma as an early indicator of hemodynamic compromise [18]. Recent obstetric studies show that SI can help predict adverse outcomes in PPH, including the need for transfusion, ICU admission, and invasive procedures, supporting its growing relevance in obstetric practice [19,20,21,22]. In this study, SI tended to be higher in the EV (+) group, and a significantly greater proportion of these patients presented with SI ≥ 1.0, suggesting that combining SI with EV may enhance prediction of the need for surgical intervention in PPH. The current WHO guideline considers a shock index ≥1 to be an abnormal hemodynamic sign in postpartum hemorrhage. Consistent with this recommendation, SI ≥1.0 was significantly more common in the EV (+) group. However, because the continuous SI did not reach statistical significance, this finding should be interpreted with caution. Additional studies are required to confirm the predictive value of this combined approach and determine its optimal clinical application in severe PPH.

To further evaluate whether contrast extravasation was independently associated with surgical management, we performed a multivariable logistic regression analysis adjusting for BMI and shock index. Contrast extravasation remained significantly associated with surgical management after adjustment for BMI and shock index, whereas BMI and shock index were not independently associated with surgical intervention. These findings support the potential value of contrast extravasation on contrast-enhanced CT as an imaging marker for identifying patients who may require more aggressive management. In addition, the multivariable logistic regression analysis should be interpreted with caution because of the limited number of surgical events and the resulting wide confidence interval for the estimated odds ratio.

This study has several limitations. Its retrospective design introduces potential selection bias, particularly regarding the decision to perform CE-CT, which may have been influenced by clinician judgment. Furthermore, because the presence of contrast extravasation was one of the clinical factors available to the treating physicians, the present study could not distinguish whether EV identified patients requiring intervention or whether the imaging finding itself influenced the decision to escalate treatment. The relatively small sample size may have limited the statistical power to detect differences in less common variables, and the fact that the study was conducted in only two centers may limit generalizability. In addition, although data were collected from two institutions to increase the study population, differences in institutional clinical practice or management strategies may have influenced the observed outcomes. Because of the limited sample size, stratified or center-adjusted analyses were not feasible, and this should be considered when interpreting our findings. Because the study period spanned more than a decade, temporal changes in CT protocols and clinical management of postpartum hemorrhage may have introduced additional heterogeneity that could not be fully accounted for in this retrospective analysis. Although radiologists were blinded to clinical outcomes, interobserver variability in the interpretation of contrast EV on CE-CT was not assessed, which may affect reproducibility. Long-term follow-up data, such as fertility outcomes, delayed complications, or quality of life measures, were not included. Despite these limitations, the study’s strength lies in the direct comparison of management outcomes between EV (+) and EV (−) groups, providing clinically relevant guidance for identifying patients who may benefit from more active management versus those suitable for conservative care.

## 5. Conclusions

The presence of contrast EV on CE-CT in patients with PPH was associated with increased need for surgical intervention, greater transfusion requirements, and higher complication rates. Additionally, a significantly higher proportion of patients with EV had a SI ≥ 1.0, suggesting early hemodynamic compromise.

## Figures and Tables

**Table 1:** Obstetric and baseline characteristics of patients with postpartum hemorrhage.

Variable	EV (−), *n* = 28	EV (+), *n* = 23	*p*-Value
Age, years	34.79 ± 4.26	34.30 ± 4.41	0.695
BMI (kg/m^2^)	26.64 ± 3.90	23.96 ± 3.88	0.021
Hypertension	3 (10.7)	1 (4.3)	0.405
Diabetes	3 (10.7)	1 (4.3)	0.405
Parity (*n*)	1.54 ± 0.88	1.61 ± 0.65	0.489
Primipara vs. Multipara	16:12 (57.1:42.9%)	12:11 (52.2:47.8)	0.723
Number of fetuses (Singleton: Multiple)	25:3 (89.3:10.7%)	21:2 (91.3:8.7)	0.811
Full-term vs. Preterm	27:1 (96.4:3.6%)	22:1 (95.7:4.3)	0.888
Type of Delivery (Vaginal vs. Cesarean)	11:17 (39.3:60.7%)	11:12 (47.8:52.2)	0.540
Previous Cesarean Delivery	7 (25%)	3 (13.0%)	0.289
Delivery location (Inborn vs. Outborn)	2:26 (7.1:92.9%)	3:20 (13.0:87.0%)	0.485
PPH onset (Primary vs. Secondary)	19:9 (67.9:32.1)	13:10 (56.5:43.5)	0.405

EV: Extravasation, BMI: Body Mass Index, PPH: Postpartum Hemorrhage.

**Table 2:** Initial blood tests and vital signs of patients with postpartum hemorrhage.

Variable	EV (−), *n* = 28	EV (+), *n* = 23	*p*-Value
Initial Blood test			
WBC (×10^3^/μL)	16.21 ± 5.70	19.34 ± 8.45	0.123
Hb (g/dL)	9.99 ± 2.02	9.53 ± 1.92	0.407
Hct (%)	29.22 ± 5.70	27.90 ± 5.51	0.406
Platelet (×10^3^/μL)	219.71 ± 86.91	232.52 ± 115.28	0.653
Lactic acid (mmol/L)	2.65 ± 1.65	2.65 ± 1.22	0.650
Initial Vital sign			
Systolic BP (mmHg)	118.25 ± 23.73	111.87 ± 24.69	0.353
Diastolic BP (mmHg)	75.04 ± 19.44	68.7 ± 18.55	0.130
Heart rate (beat/min)	89.57 ± 14.75	101.61 ± 27.55	0.052
Body temperature (°C)	37.21 ± 0.92	37.00 ± 0.66	0.369
Shock index	0.78 ± 0.22	0.95 ± 0.37	0.062
Shock index ≥ 1.0 (%)	3 (10.7)	9 (39.1)	0.017

EV: Extravasation, WBC: White Blood cell, Hb: hemoglobin, Hct: Hematocrit, BP: Blood pressure.

**Table 3:** Causes of postpartum hemorrhage.

Variable	EV (−), *n* = 28	EV (+), *n* = 23	*p*-Value
Cause			0.496
Tone	18 (64.3%)	11 (47.8%)	
Trauma	6 (21.4%)	7 (30.4%)	
Tissue	4 (14.3%)	5 (21.7%)	
Thrombin	0 (0%)	0 (0%)	

EV: Extravasation.

**Table 4:** Management strategies for postpartum hemorrhage.

Variable	EV (−), *n* = 28	EV (+), *n* = 23	*p*-Value
Management Strategy			0.001
Conservative management only (%)	25 (89.3%)	10 (43.5%)	
Conversion to Surgery (%)	2 (7.1%)	3 (13.0%)	
Immediate surgical management (%)	1 (3.6%)	10 (43.5%)	
Overall surgical management (%)	3 (10.7%)	13 (56.5%)	
Type of Surgical Management			
Laceration Suture	0 (0%)	2 (8.7%)	
Placental Removal	0 (0%)	1 (4.3%)	
Hysterectomy	2 (7.1%)	7 (30.4%)	
Other surgical procedures	1 (3.6%)	3 (13.0%)	
Type of conservative management			
Uterotonics	19 (67.9%)	13 (56.5%)	0.405
Hemostatics	16 (57.1%)	12 (52.2%)	0.723
Curettage (evacuation)	1 (3.6%)	0 (0%)	0.365
Gauze Packing	3 (10.7%)	4 (17.4%)	0.495
Intrauterine Balloon Tamponade	6 (21.4%)	3 (13.0%)	0.439
Uterine Artery Embolization	8 (28.6%)	11 (47.8%)	0.157

EV: Extravasation.

**Table 5:** Outcomes of postpartum hemorrhage.

Variable	EV (−) (*n* = 28)	EV (+) (*n* = 23)	*p*-Value
Total EBL (mL)			0.009
500–1000 (%)	18 (64.3%)	8 (34.8%)	
1000–2000 (%)	9 (32.1%)	7 (30.4%)	
>2000 (%)	1 (3.6%)	8 (34.8%)	
Admission			
Length of Hospital stay(days)	4.61 ± 2.61	6.88 ± 3.78	0.034
ICU admission	3 (10.7%)	8 (34.8%)	0.039
Blood Transfusion			
Packed RBC (packs)	2.54 ± 2.21	7.61 ± 7.59	0.002
FFP (packs)	1.75 ± 2.33	7.43 ± 8.34	0.002
PC (packs)	1.71 ± 4.70	11.26 ± 16.02	0.005
Complications			
Mortality	0	0	N/A
DIC	2 (7.1%)	7 (30.4%)	0.032
ARF	0	0	N/A
Fever	6 (21.4%)	3 (13.0%)	0.439
Hypertension	0 (0%)	3 (13.0%)	0.051
Pulmonary Complications	0 (0%)	3 (13.0%)	0.051
Cardiac Complications	0 (0%)	1 (4.3%)	0.270
Cerebral Complication	0 (0%)	1 (4.3%)	0.270
DVT	0 (0%)	0 (0%)	N/A
Sheehan Syndrome	0 (0%)	0 (0%)	N/A

EV: Extravasation, EBL: Estimated Blood Loss, ICU: Intensive Care Unit, RBC: Red Blood Cell, FFP: Fresh Frozen Plasma, PC: Platelet Concentrate, DIC: Disseminated Intravascular Coagulation, ARF: Acute Renal Failure, N/A: not applicable, DVT: Deep Vein Thrombosis.

**Table 6:** Multivariable logistic regression analysis for factors associated with overall surgical management.

Variable	Regression Coefficient	SE	Adjusted OR	95% CI	*p*-Value
EV	2.007	0.790	7.444	1.582–35.033	0.011
BMI	−0.125	0.104	0.882	0.719–1.082	0.229
SI ≥ 1.0	0.887	0.831	2.428	0.4777–12.368	0.285

SE: standard error, OR: odds ratio, CI: confidence interval, EV: extravasation, BMI: body mass index, SI: shock index.

**Table 7:** Summary of key studies on the utility of CE-CT in PPH.

Author (Year)	Study Design/Sample	Image Modality	Main Findings	Clinical Implication
Lee et al., (2010) [4]	Retrospective *n* = 27	MDCT	- Active bleeding site correctly identified with 96% concordance to angiography. - Sensitivity 100%, specificity 96%, accuracy 97%.	MCDT may have a role in the detection and localization of PPH and may yield Supplementary Information on extrauterine abnormalities.
Kim et al. (2020) [6]	Retrospective *n* = 57	MCDT	- EV detected in 33 cases. - EV group: higher blood loss (2100 vs. 1170mL), more transfusion (6 vs. 3 units), higher UAE rate (64% vs. 8%).	CE-MDCT is helpful to determine which patients are candidates for UAE.
Mitoma et al. (2024) [7]	Retrospective *n* = 60	Dynamic CT (early/late phase)	- Early phase CME was associated with UAE performance, with a sensitivity of 95%, specificity of 87%, positive predictive value of 80%, and negative predictive value of 97%.	UAE is not required when CME cannot be detected in the uterine cavity. If early phase CME is present, UAE should be considered immediately.
Suzuki et al. (2024) [8]	Retrospective *n* = 150	Dynamic CE-CT	- EV observed in 53%. - Proposed “PRACE’ concept. - PRACE cases required massive transfusion and invasive procedures.	CE-dCT plays a pivotal role in elucidating the etiology of PPH and guiding therapeutic interventions.
Kawamura et al. (2014) [11]	Prospective *n* = 26	Dynamic CT	- EV found in 46%. - Enabled localization of upper uterine or vaginal arterial bleeding. - Negative EV extracted cases of PPH that were well controlled without the need for surgical or radiologic intervention.	Dynamic CT aids in identifying focal arterial bleeding and guides balloon tamponade or UAE, reducing need for hysterectomy.
Jung et al. (2020) [12]	Retrospective *n* = 61	CT angiography	- EV detected in 61%. - Among EV (+), 78% underwent UAE; 83% confirmed active bleeding on angiography. - EV (+) group higher transfusion volume and massive transfusion rate (27% vs. 0%).	CT angiography reliably predicts the need for UAE; EV (−) patients can often avoid invasive procedures.

PPH: postpartum hemorrhage, CE-CT: contrast-enhanced computed tomography, CE-dCT: dynamic contrast-enhanced CT, MDCT: multidetector Computed Tomography, CTA: Computed tomography angiography, EV: extravasation, CME: contrast medium extravasation, UAE: uterine artery embolization, PRACE: Postpartum hemorrhage resistance to treatment, and arterial contrast extravasation.

## Data Availability

Data are available from the authors upon reasonable request.
